# Impact of an Ambient AI Scribe Among Clinicians and Patients: Real-World Prospective Observational Time-Motion Study

**DOI:** 10.2196/85580

**Published:** 2026-03-31

**Authors:** Jonathan Yue En Tan, Iffat Bin Mohammad Rafi, Gerald Gui Ren Sng, Joshua Yi Min Tung, Daniel Yan Zheng Lim, Jasmine Chiat Ling Ong, Gek Hsiang Lim, Eileen Yi Lee Lew, Kuok Wei Chia, Mark Kok Hong Heng

**Affiliations:** 1 Department of Future Health System, Singapore General Hospital, 1 Hospital Blvd, SingHealth TowerSingapore, Singapore; 2 Data Science and Artificial Intelligence Laboratory, Singapore General Hospital, Singapore, Singapore; 3 Department of Endocrinology, Singapore General Hospital, Singapore, Singapore; 4 Department of Urology, Singapore General Hospital, Singapore, Singapore; 5 Department of Gastroenterology and Hepatology, Singapore General Hospital, Singapore, Singapore; 6 Division of Pharmacy, Singapore General Hospital, Singapore, Singapore; 7 Duke-NUS AI + Medical Sciences Initiative, Duke-NUS Medical School, Singapore, Singapore; 8 Health Services Research Unit, Singapore General Hospital, Singapore, Singapore; 9 Office of Insights and Analytics, SingHealth, Singapore, Singapore

**Keywords:** ambient intelligence, generative artificial intelligence, clinical documentation, physician-patient interaction, time-motion study, eye contact, ambient scribe, artificial intelligence, AI

## Abstract

**Background:**

Ambient artificial intelligence scribes are increasingly used to reduce clinician burnout and cognitive load, although their impact on documentation time remains inconsistent across studies. Most existing real-world impact studies have been conducted in the United States and rely on electronic health record time stamps, which may not accurately reflect actual documentation time.

**Objective:**

This study aimed to quantify the impact of ambient scribe technology on clinician efficiency and patient engagement in an outpatient setting using direct observational methods and patient surveys.

**Methods:**

We conducted a prospective, within-clinician quality improvement study at a large academic medical center in Singapore from December 2024 to May 2025. Nine clinicians participated in matched observation sessions with and without an in-house ambient scribe tool. Five trained observers used standardized time-motion methodology to capture documentation time, proportion of eye contact, and consultation duration across 169 consultations. Patient surveys were administered following consultations using the ambient scribe. Linear mixed-effects models were used to account for clustering within clinicians.

**Results:**

Ambient scribe use was associated with a 15.0% reduction in documentation time per consultation (mean 5.3, SD 2.7 minutes to 4.5, SD 2.4 minutes; *P*=.04); a 10.6% increase in proportion of eye contact time (mean 69.6%, SD 18.6% to 77.1%, SD 17.7%; *P*=.009); and no significant change in consultation duration (mean 11.5, SD 6.9 minutes vs 10.9, SD 5.6 minutes; *P*=.42) or total cycle time per patient (mean 13.7, SD 7.7 minutes vs 13.2, SD 6.3 minutes; *P*=.57), where total cycle time includes preclerking, consultation, and postconsultation documentation. Effects were consistent across new and follow-up patients. Patient acceptance was favorable: of 39 surveyed patients, 27 (69.2%) agreed that their physician focused on them more during the consultation, and none expressed discomfort with the technology.

**Conclusions:**

This evaluation of ambient scribe use in an Asian health care setting demonstrates that experienced users achieved reduced documentation time and improved patient engagement without affecting consultation duration or total time per patient. The technology was also well accepted by patients. Taken together, these findings suggest that ambient scribes reallocate clinician effort toward patient interaction rather than enabling faster patient turnover, supporting their implementation across diverse health care settings.

## Introduction

Ambient scribes use artificial intelligence (AI) to convert verbal clinician-patient interactions into structured clinical documentation. This is one of the fastest-adopted uses of generative AI in health systems worldwide [1].

Recent real-world studies across health systems in the United States demonstrate consistent improvements in clinician well-being and patient interactions with the use of ambient scribes, with studies reporting significant reductions in cognitive load, task burden, and burnout scores alongside enhanced patient experience during consultations [2-7]. However, reported time savings vary considerably between organizations. While some implementations report substantial reductions in documentation time and after-hours charting, others show minimal productivity gains [8,9].

To our knowledge, no comprehensive real-world impact studies have been conducted outside of the United States, leaving questions about the technology’s effectiveness in other health care contexts unanswered. Documentation burdens and clinical workflows may differ between countries, potentially affecting the magnitude of time savings and productivity gains achievable through the use of clinical ambient scribes. Additionally, no published studies have evaluated clinical ambient scribe outcomes in multilingual clinical environments despite the prevalence of such settings in many health care systems worldwide. Shah et al [7] have previously highlighted limited functionality with non–English-speaking patients as a specific barrier to ambient scribe adoption. As digital health tools increasingly permeate global health care systems, understanding their implementation in linguistically diverse environments is important for ensuring equitable and effective deployment.

From a methodological perspective, existing studies have relied primarily on electronic health record (EHR) time stamp analysis to measure documentation time and productivity outcomes [4-6,10]. While convenient for large-scale analysis, EHR-based metrics provide only proxy measures that may not accurately reflect the actual time spent on documentation tasks. These metrics often rely on time stamps that capture when a note window is open, which may overestimate documentation time if clinicians multitask or leave notes open [11]. Direct time-motion observation, though more resource intensive, offers superior accuracy in distinguishing active documentation from passive or multitasking periods [12].

To address these evidence gaps, we aimed to determine the effectiveness and acceptability of ambient scribe technology when compared to the standard of care in a multilingual health care setting using direct observational methods.

## Methods

### Study Design

We conducted a prospective within-clinician observational study to evaluate the real-world clinical impact of an in-house ambient AI scribe in outpatient clinics of Singapore General Hospital, a quaternary referral center with more than 1700 beds that is the largest academic medical center in Singapore [13]. This study was conducted over 5 months between December 27, 2024, and May 30, 2025. It builds on a previous pilot study at the same institution that demonstrated the ambient scribe tool’s ability to generate high-quality clinical notes comparable to those written by clinicians [14].

### Ambient Scribe Technology

Note Buddy, the ambient scribe tool used in this study, was developed in-house using automatic speech recognition and large language model technology. This tool supports transcription in 5 languages commonly used in Singapore: English, Mandarin, Malay, Tamil, and Cantonese. However, during the study period, only English, Mandarin, and Malay consultations were observed.

**Figure 1.**
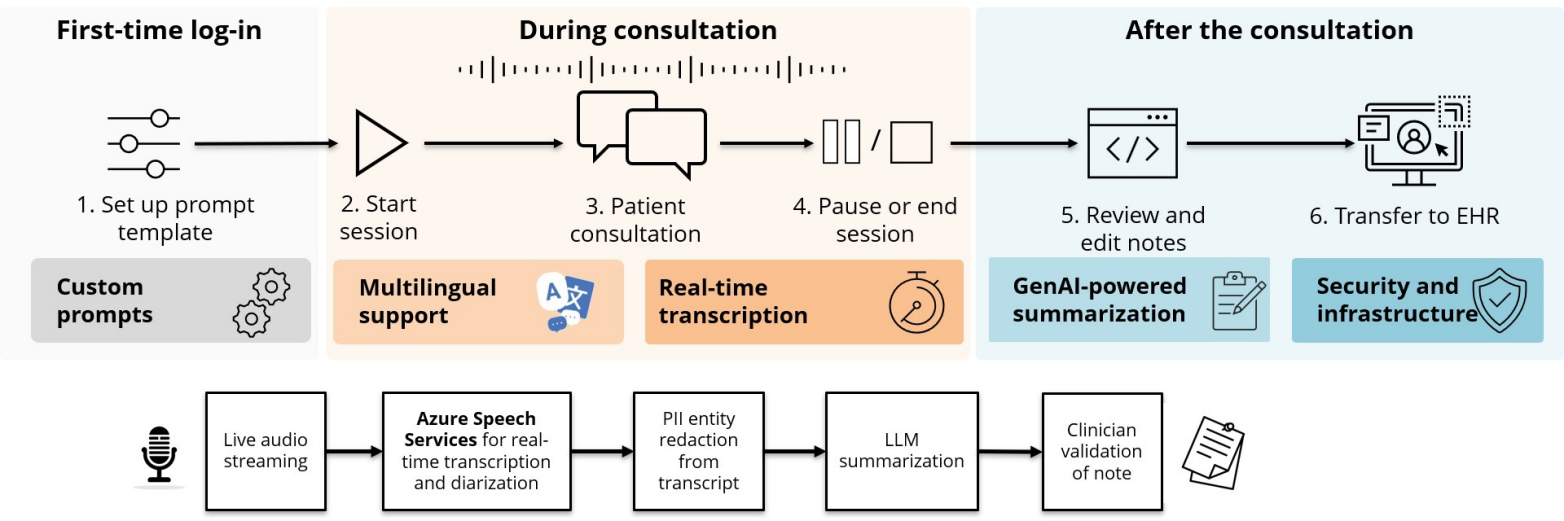
Workflow of Note Buddy use in clinical practice. Users configure prompt templates that specify the structure and formatting of the generated notes. During consultations, the system transcribes clinician-patient interactions in the supported languages. After each consultation, the ambient scribe processes the transcript using the predefined prompt to generate a draft note, which clinicians review, edit if needed, and manually transfer into the electronic health record (EHR). GenAI: generative artificial intelligence; LLM: large language model; PII: personally identifiable information.

Note Buddy can process conversations of up to approximately 96,000 words (based on token limits), identifying speakers and languages at the word level. It leverages GPT-4o’s (OpenAI) native multilingual capabilities to process mixed-language transcripts without pretranslation or model fine-tuning. Note Buddy then reformats and summarizes transcripts into clinical consultation notes. On the basis of routine system use, note generation (from end of consultation to completed draft) typically requires 10 to 30 seconds, with duration varying by consultation length and prompt complexity. The system processes audio in real time without storing recordings (Figure 1).

Users can customize the generated notes by creating prompt templates specifying note structure and clinical terminology, providing example notes for guidance, or selecting from preconfigured prompts in the prompt library. As the tool was not integrated into the EHR during the study period, clinicians had to manually transfer the AI-generated notes from the ambient scribe into the EHR.

### Participants and Setting

This study was conducted in an outpatient clinic setting where consultations naturally occur in multiple languages according to patient preferences. We recruited clinicians who had generated at least 30 notes using the ambient scribe tool in the 3 months prior to study commencement.

Clinicians unable to participate in 2 consecutive clinic sessions were excluded. Due to the time-intensive nature of direct observation, we intentionally focused on experienced clinician users who had achieved proficiency with the system and were most likely to demonstrate its optimal impact. From 27 eligible clinicians, 9 (33.3%) were selected through convenience sampling based on scheduling feasibility and observer availability.

Each clinician selected 2 clinic sessions for observation: one session using the ambient scribe tool and one using standard documentation methods. Clinicians were asked to select sessions with a similar patient case mix and clinic characteristics. Standard documentation methods consisted of manual typing, with some clinicians potentially using built-in EHR features such as smart phrases. The use of such features was not systematically recorded. Each clinician decided whether to use the ambient scribe tool in the first or second observed session; this was not randomized.

The interval between the 2 clinic sessions was kept within 2 weeks to minimize changes in practice patterns over time. Scheduled appointments remained unchanged to reflect the natural distribution of new and follow-up patients in the real-world setting. The total number of patients per clinic session ranged from 8 to 15.

### Data Collection: Observation Methodology

Five observers carried out the observations, with each observer consistently assigned to the same clinician across both clinic sessions. All observations were conducted in person, with the observer present in the consultation room during scribe and nonscribe sessions.

All 5 observers participated in a joint training session to minimize interobserver variability. This included instruction on using a macro-enabled Microsoft Excel worksheet for time-motion data collection. The session concluded with group practice on mock consultations until a consensus was reached on measurement definitions and recording techniques.  

**Figure 2.**
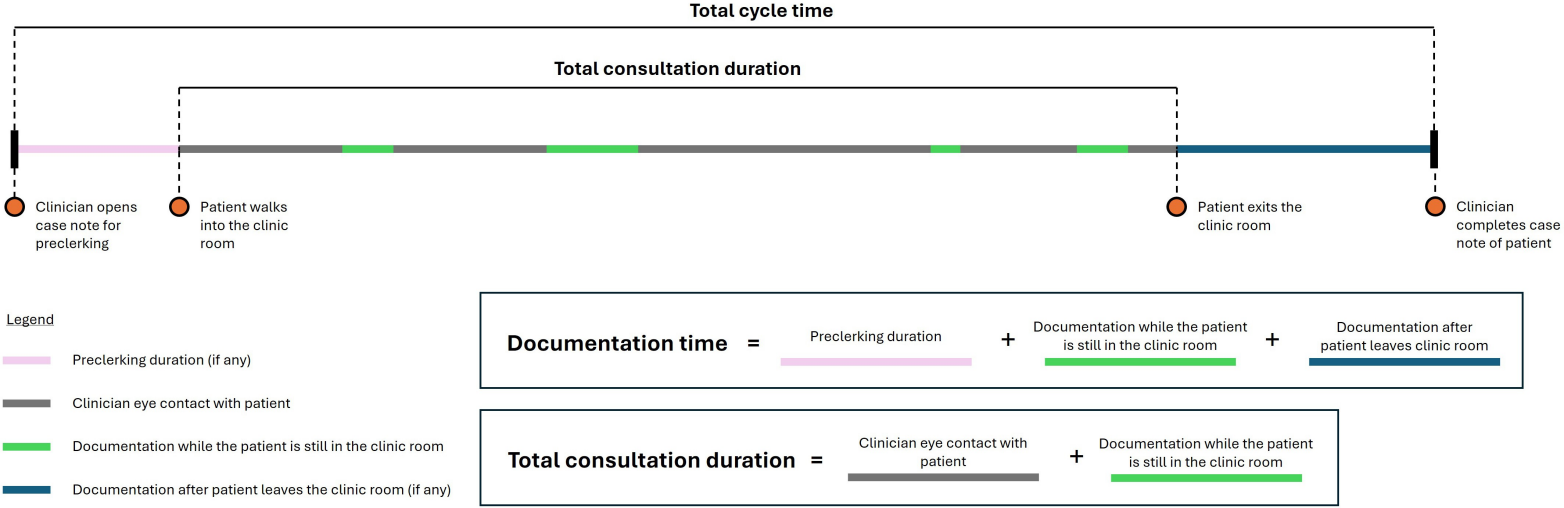
Schematic representation of time components measured in the observational time-motion study. Documentation time is the time taken for documentation performed before, during, or immediately after the consultation, defined as the total time the clinician interacted with the computer. Consultation duration is the time between patient entry and exit from the consultation room. Total cycle time is derived from the start of preclerking (if any) to completion of documentation. All time points were recorded in real time by trained observers using a standardized macro-enabled Microsoft Excel worksheet.

Using the standardized macro-enabled Microsoft Excel worksheet, observers recorded real-time data on 3 key metrics and 1 derived metric. Consultation duration was calculated as the time between patient entry and exit from the consultation room. Proportion of eye contact was defined as the percentage of the consultation duration for which the clinician was looking directly at the patient. Documentation time was measured as the total time that the clinician interacted with the computer across all phases of the patient encounter: preclerking (if any), during the consultation, and immediately after the consultation, as illustrated in Figure 2.

Total cycle time was calculated as a derived metric from preclerking start (if any) to documentation completion, as illustrated in Figure 2. This represents the complete time investment per patient encounter and directly reflects potential impact on clinic throughput.

As this ambient scribe was designed for real-time documentation, observers recorded documentation completed within the observation window, which required clinicians to finish notes during or immediately after consultations rather than deferring to later periods. In the sessions with ambient scribe use, documentation time included the time spent reviewing, editing, and manually transferring AI-generated notes into the EHR, as well as the time taken for the ambient scribe to generate the note. Observers did not separately track the time spent on each component.

### Data Collection: Sample Size Explanation

Due to the resource-intensive nature of direct observation and the pragmatic evaluation design, we determined the sample size based on clinician availability and feasibility constraints rather than formal power calculations. A post hoc approximation using 2-tailed paired *t* test assumptions (effect size=0.5; α=.05; power=0.95) indicated a minimum of 54 consultations per group (total 108 consultations). This simplified calculation does not account for the clustering of consultations within clinicians, which is addressed by our mixed-effects models. It serves as a conservative proxy given the complexity of power calculations for such models. Our final sample of 169 consultations exceeded this threshold.

### Patient Surveys

Patient surveys were conducted to evaluate patient perspectives on ambient scribe use. Four of the 9 participating clinicians were selected through convenience sampling for patient surveys based on surveyor availability and clinician consent. Surveys were conducted across selected clinic sessions, resulting in partial overlap with time-motion observations, although the 2 data collection activities were conducted independently. Surveys were administered verbally by independent surveyors from the Office of Patient Experience immediately following consultations in which the ambient scribe tool was used. The survey assessed 4 domains: perceived quality of the clinical encounter, clinician’s attentiveness and engagement, comfort level with AI-assisted documentation, and likelihood of recommending the technology for other clinical encounters. Patients were excluded from surveys if they declined participation or were deemed clinically unsuitable by their physician, for example, due to having just received a poor prognosis.

### Statistical Analysis

We used descriptive statistics to summarize the characteristics of participating clinicians and consultation sessions. We applied the Pearson chi-square test to assess whether the distribution of new vs follow-up patients differed between consultations conducted with and without the ambient scribe tool. Linear mixed-effects models were used to account for clustering of consultations within individual clinicians and unequal numbers of observations per clinician. These models included random intercepts for individual clinicians to control for baseline differences in practice patterns and fixed effects for ambient scribe use and patient type (new vs follow-up consultation). Statistical significance was set at *P*<.05, and all analyses were conducted using SPSS Statistics (version 26.0; IBM Corp).

This manuscript was prepared using the SQUIRE (Standards for Quality Improvement Reporting Excellence) reporting guidelines.

### Ethical Considerations

This study evaluated process metrics of an existing ambient scribe tool without collecting identifiable patient data or implementing clinical interventions. In accordance with institutional governance policies, the study was determined to constitute a quality improvement initiative and, therefore, did not require formal institutional review board approval (SingHealth Policy for Governance on Data Use [Non-Research]; document no. SHS-DDS-DAG-1002 [internal policy document]). For routine clinical use of the ambient scribe, explicit patient consent was not required based on institutional guidance as the system functions solely as a documentation support tool and does not store audio recordings. No audio recordings, transcripts, or personally identifiable information were retained for evaluation purposes, and analyses were limited to deidentified workflow and time-motion metrics. Verbal consent was obtained from patients participating in anonymous satisfaction surveys. Participation was voluntary, and no financial or material compensation was provided to clinicians or patients.

## Results

### Sample Characteristics

Nine clinicians participated in this study, representing 7 different specialties (Table 1). They had a mean of 20.8 (SD 7.8) years in practice. Each clinician completed matched observation sessions with and without ambient scribe use. Of the 9 clinicians, 4 completed their first observed session using the ambient scribe, and 5 completed their first session without it, resulting in an approximately even distribution of session order. In this study, all clinicians used their own preconfigured prompts and did not make any further modifications to the prompts.

**Table 1.** Characteristics of participating clinicians in the observational study of ambient scribe use (n=9).

Characteristics	Values
Duration in practice (y), mean (SD)	20.8 (7.8)
Observed consultations per clinician, mean (SD)	18.8 (6.5)
Clinicians per specialty, n	
Respiratory medicine	2
Endocrinology	2
Gastroenterology	1
Neurology	1
Rehabilitation medicine	1
Neonatology	1
General surgery	1

As shown in Table 2, a total of 169 consultations were observed across the study period: 78 (46.2%) conducted with the ambient scribe and 91 (53.8%) conducted without it. The unequal distribution reflected natural variation in scheduled appointments and patient attendance across the matched clinic sessions. Of these 169 consultations, 54 (32.0%) involved new patients, and 115 (68.0%) were follow-up appointments. Of the 78 consultations with ambient scribe use, 63 (80.8%) were in English, 14 (17.9%) were in Mandarin, and 1 (1.3%) was in Malay.

**Table 2.** Distribution of patient types (new vs follow-up) among consultations conducted with and without the artificial intelligence (AI) scribe. The proportions of patient types were similar across groups, with no statistically significant difference in distribution.

Patient type	Overall (n=169), n (%)	Without AI scribe use (n=91), n (%)	With AI scribe use (n=78), n (%)	*P* value^a^
New patients	54 (32.0)	28 (30.8)	26 (33.3)	.72
Follow-up patients	115 (68.0)	63 (69.2)	52 (66.7)	.72

a Chi-square test comparing distribution between groups.

**Figure 3.**
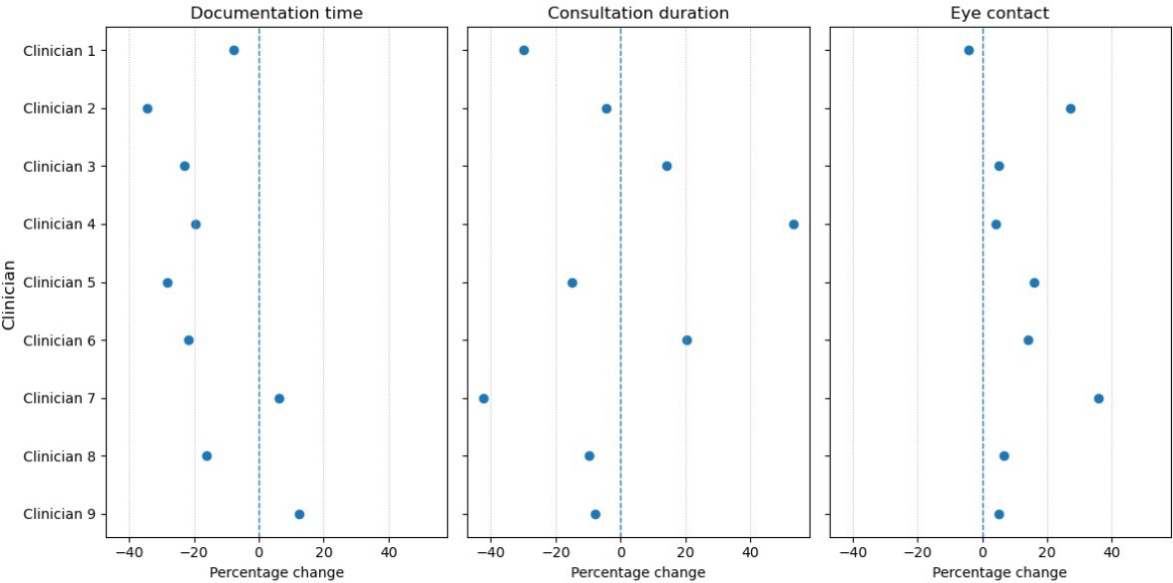
Unadjusted clinician-level outcome changes with artificial intelligence (AI) scribe use. Each dot represents the percentage change for an individual clinician comparing sessions with and without AI scribe use. The dashed line indicates no change (0%).

Given the potential for variation in individual clinician response, we examined unadjusted clinician-level changes comparing sessions with and without ambient scribe use (Figure 3). Substantial heterogeneity was observed: 7 of 9 clinicians demonstrated reductions in documentation time (7.9%‐34.6%), 6 of 9 had shorter consultation durations (4.3%‐42.2%), and 8 of 9 showed increased eye contact (4.2%‐35.8%).

Table 3 summarizes the outcomes associated with the use of ambient scribes. Use of the tool was associated with 15.0% less time spent on documentation (from mean 5.3, SD 2.7 minutes to mean 4.5, SD 2.4 minutes per consultation; *P*=.04). The proportion of eye contact was 10.6% higher with ambient scribe use compared with standard documentation (from mean 69.6%, SD 18.6% to mean 77.1%, SD 17.7% of consultation duration; *P*=.009). Consultation duration was similar between conditions (mean 11.5, SD 6.9 minutes vs mean 10.9, SD 5.6 minutes; *P*=.42), and total cycle time per patient also did not differ significantly (mean 13.7, SD 7.7 minutes vs mean 13.2, SD 6.3 minutes; *P*=.57). The ambient scribe effects were consistent across new and follow-up patients for both documentation time and eye contact (interaction *P* values of .79 and .35, respectively), indicating uniform benefits across different consultation types.

**Table 3.** Comparison of outcomes between consultations conducted with and without artificial intelligence (AI) scribe use. Adjusted differences were calculated using linear mixed-effects models accounting for clinician-level clustering.

Outcome	Without AI scribe use (unadjusted), mean (SD)	With AI scribe use (unadjusted), mean (SD)	Adjusted difference (95% CI)	Change (%)	*P* value
Documentation time per consultation (min)	5.3 (2.7)	4.5 (2.4)	−0.8 (−1.5 to –0.05)	−15.0	.04
Proportion of eye contact time (%)	69.6 (18.6)	77.1 (17.7)	+7.4 (1.9 to 13.0)	10.6	.009
Consultation duration (min)	11.5 (6.9)	10.9 (5.6)	−0.7 (−2.6 to 1.1)	−6.1	.42
Total cycle time (min)	13.7 (7.7)	13.2 (6.3)	−0.6 (−2.6 to 1.4)	−4.4	.57

Crude independent-sample *t* tests treating all consultations as independent observations yielded similar effect sizes for eye contact (+7.5%; *P*=.008) and consultation duration (–0.67 minutes; *P*=.50). However, the reduction in documentation time reached statistical significance only in the adjusted mixed-effects analysis (crude: −0.74 minutes and *P*=.07; adjusted: –0.8 minutes and *P*=.04). This highlights the importance of accounting for clinician-level clustering.

### Secondary Outcomes: Patient Experience

Patient surveys were conducted across 4 clinic sessions involving 4 of the 9 participating clinicians. Of 45 eligible patients in these sessions, 39 (86.7%) completed the surveys; 6 (13.3%) declined participation or were deemed unsuitable by their physician. Of the 39 surveyed patients, 18 (46.2%) were also included in the time-motion observations.

Age demographics are shown in Table 4, with 41.0% (16/39) of the patients aged 65 years and above. Survey language preferences were English (31/39, 79.5%), Mandarin and Chinese dialects (7/39, 17.9%), and Malay (1/39, 2.6%). Survey responses were collapsed into 3 analytic categories (“yes,” “neutral,” and “no”) based on evaluative meaning. Positive responses were coded as “yes,” neutral or indeterminate responses were coded as “neutral,” and negative responses were coded as “no.” The complete survey instrument is detailed in Multimedia Appendix 1.

**Table 4.** Age distribution of patients who completed surveys about ambient scribe use during consultations.

Age group (y)	Patients (n=39), n (%)
0‐19	1 (2.6)
20‐29	3 (7.7)
30‐39	7 (17.9)
40‐49	5 (12.8)
50‐64	7 (17.9)
≥65	16 (41.0)

**Figure 4.**
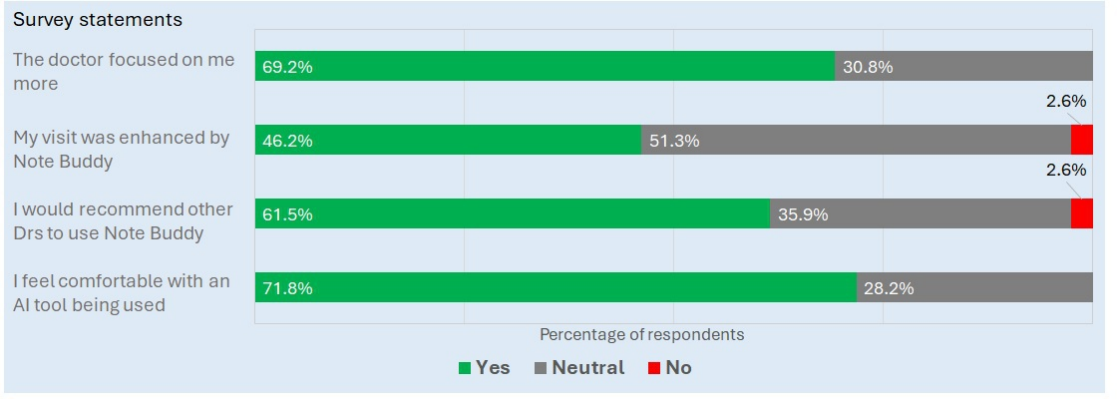
Patient perceptions of ambient artificial intelligence (AI) scribe use (Note Buddy). Postconsultation patient surveys assessed perceived clinician attentiveness, consultation experience, comfort with AI-assisted documentation, and willingness to recommend the technology. Responses were collapsed into “yes,” “neutral,” and “no” categories for presentation.

Overall, patient acceptance of the AI tool was favorable (Figure 4), with 69.2% (27/39) agreeing that their physician focused on them more during the consultation and 61.5% (24/39) expressing willingness to recommend the technology for use by other physicians. While some patients (20/39, 51.3%) gave neutral responses and 2.6% (1/39) reported that the ambient scribe did not enhance their visit, none expressed discomfort with its use.

Analysis of the overlapping subset of patients who were both surveyed and included in time-motion observations (18/39, 46.2%) showed internal consistency between objective measurements and patient perceptions: 61.1% (11/18) of these patients reported that their physician focused on them more during the consultation, whereas measured eye contact was 15.2% higher compared to the same clinicians’ sessions without the ambient scribe (mean 72.7%, SD 15.9% vs mean 63.1%, SD 16.9%; *P*=.07). This directional alignment between objective and subjective measures supports the validity of both metrics.

To assess potential selection bias in the patient survey data, we examined whether the effect of the ambient scribe differed between clinicians whose patients were surveyed and those whose patients were not. After adjusting for patient type (new vs follow-up), there was no evidence of a differential effect for either documentation time (interaction *P*=.71) or proportion of eye contact (interaction *P*=.62). These findings suggest that the patient survey results are representative of the broader clinician cohort.

## Discussion

### Principal Findings

To our knowledge, this is the first study of the real-world impact of an ambient scribe conducted in Asia. Use of an in-house ambient scribe tool was associated with reduced documentation time and increased eye contact, whereas consultation duration remained unchanged. This suggests that clinicians redirected time from clerical tasks toward patient engagement rather than shortening consultations. The overlap between surveyed patients and time-motion observations (18/39, 46.2% of patients) provides supportive internal consistency, with patients who experienced objectively measured increases in eye contact also reporting improved clinician attentiveness.

Allowing clinicians to choose in which clinic session to use the ambient scribe instead of randomizing may have introduced selection bias. However, patient mix and volume were comparable between the scribe and nonscribe sessions (ratio of new to follow-up patients: *P*=.72; patient volume per session: mean 9.0, SD 3.4 with scribe use vs mean 9.8, SD 4.1 without scribe use; *P*=.44), suggesting that any such bias was minimal.

Our findings align with those of recent US studies showing that time savings tend to be modest. Documentation time was estimated to have been reduced by 0.8 minutes (95% CI −1.5 to −0.05; *P*=.04) in the intervention group. Although the upper confidence limit approached zero, indicating potential sensitivity to individual outliers, our observed reduction of 0.8 minutes per consultation is consistent with that observed in prior observational studies. These include findings from Sutter Health (drop from 6.2 to 5.3 minutes) [6], The Permanente Medical Group (0.4-minute reduction) [15], and Stanford Health Care (0.57-minute reduction) [10], as well as recent randomized controlled trials reporting similar results [4,16]. Additionally, our study uniquely demonstrates improved patient engagement through increased eye contact, which we were able to quantify because we used direct observation rather than EHR-based metrics.

Unsurprisingly, this real-world evaluation demonstrated heterogeneity in individual-level effects between clinicians. However, our mixed-effects model was specifically designed to account for this individual variation while estimating the overall treatment effect, which remained statistically significant despite the heterogeneity. This within-clinician comparison design controlled for individual clinician variability and practice patterns that could confound results. Although our linear mixed-effects model relied on only 9 clinician clusters, which can affect the reliability of variance estimates and increase the risk of type 1 errors in mixed-model analyses, this design allowed us to isolate the specific effects of ambient scribe implementation while accounting for the natural variation in consultation styles and documentation practices across specialists.

Our findings also demonstrate that benefits observed in Western health care systems can be realized in a different cultural and linguistic context. Of the 78 consultations that used the ambient scribe, 63 (80.8%) were conducted in English, 14 (17.9%) were conducted in Mandarin, and 1 (1.3%) was conducted in Malay, highlighting the tool’s utility in multilingual environments. The favorable patient responses observed, particularly among older adults, who comprised 41% (16/39) of survey respondents, provide reassuring evidence of acceptance across age groups.

The reproducibility of modest time savings across studies—including our observed 0.8-minute reduction—raises important questions about clinical meaningfulness at the individual clinician and health system levels. Notably, our finding that consultation duration remained unchanged despite reduced documentation time indicates that the time savings did not result in shorter patient encounters. Furthermore, our finding that total cycle time also remained unchanged demonstrates that ambient scribes should not be expected to increase clinic throughput or patient volume at the system level. Recent randomized controlled studies suggest that the primary value of this technology lies in reducing clinician burnout [4,16]. Our results complement this by demonstrating measurable improvements in patient engagement through increased eye contact and positive patient survey responses. Taken together, these findings suggest that ambient scribes should be viewed primarily as quality-of-care interventions that shift clinician attention from clerical tasks to patient interaction rather than as productivity tools [15,17].

### Limitations

This study has several limitations. First, the sample was limited to 9 clinicians in specialist outpatient clinics due to the resource-intensive nature of direct observation, which restricts generalizability to other specialties and primary care settings. We focused on experienced ambient scribe users to reflect real-world use patterns as prior research shows that regular users account for 89% of system activations [18]. This means that our findings reflect optimal rather than average implementation outcomes and cannot predict outcomes during initial adoption or among infrequent users. Because our clinicians were both experienced scribe users and relatively senior (mean 20.8, SD 7.8 years of practice), we cannot determine whether the observed benefits reflect scribe proficiency or preexisting EHR documentation efficiency. If our senior clinicians had already optimized their workflows through customized EHR templates and shortcuts, the observed time savings may underestimate the potential benefits for less efficient documenters. Conversely, new users may experience a learning curve that could temporarily reduce initial gains.

Second, the ambient scribe tool required manual transfer of notes into the EHR, which likely underestimates potential time savings compared to fully integrated systems and limits direct comparability with studies of integrated solutions.

Third, several potential biases may have influenced our findings. Clinicians self-selected the sessions in which they used the ambient scribe, introducing the possibility of selection bias. Observer expectancy and Hawthorne effects [19] may have influenced clinician behavior or the measurement of eye contact as observers and clinicians could not be blinded. Interrater reliability could not be assessed because multiple observers in the consultation room would have been intrusive; however, each clinician was consistently observed by the same individual across both conditions to minimize observer-related variability. Patient survey responses may have also been subject to social desirability bias as patients may have been reluctant to express negative views about the technology when surveyed immediately following their consultation within the clinical facility despite the use of independent surveyors. As the ambient scribe tool was developed in-house, some degree of institutional bias is possible, although objective measurement methods were used to minimize this risk.

Finally, this study did not specifically assess whether note accuracy was preserved in the context of the observed reductions in documentation time; nonetheless, the tool’s clinical fidelity had been reported in a previous study [14]. The predominance of English-language consultations (63/78, 80.8%) limits conclusions about multilingual performance. Clinicians used different prompts, including customized ones, and we did not systematically analyze variation in prompt length or complexity. Such prompt variability may have influenced the time taken for the ambient scribe to generate a note and represents a potential confounder.

### Future Directions

The observed heterogeneity in individual-level effect sizes in this real-world study highlights that the potential benefits of ambient scribe adoption may not be uniform even in experienced users. Future studies should evaluate the impact of ambient scribe technology across a broader range of clinicians and practice environments to identify which user profiles benefit the most and which may require additional support. Qualitative research into factors affecting adoption and sustained use will be important for ensuring long-term effectiveness and scalability. Future work should systematically evaluate how prompt design—including specialty-specific or individualized prompts—affects the quality of AI-generated documentation and the time required for clinician review and editing. While our study suggested effectiveness in a multilingual patient population, most consultations (63/78, 80.8%) were conducted in English. Further work is needed to assess whether the same benefits are observed in non–English-language consultations as language may influence both scribe performance and outcomes.

### Conclusions

This quality improvement study found that ambient scribe use among experienced users was associated with modest reductions in documentation time and improved patient engagement, with high patient acceptance. These findings quantify the impact of ambient scribes in a real-world outpatient setting using direct observational methods. Our findings demonstrate that these benefits can be achieved through in-house–developed solutions, offering health care systems an alternative to commercial products in cases in which data privacy, cost, or localization are key concerns.

## Supplementary material

10.2196/85580 Multimedia Appendix 1 Supplementary materials, including technical details on the ambient artificial intelligence scribe (Note Buddy), the time-motion study instrument, and the patient survey instrument.

## Abbreviations

AI: artificial intelligence
EHR: electronic health record
SQUIRE: Standards for Quality Improvement Reporting Excellence
