# Supplementary material for: Impact of an Ambient AI Scribe Among Clinicians and Patients: Real-World Prospective Observational Time-Motion Study
Source: JMIR Med Inform. 2026 Mar 31;14:e85580. doi: 10.2196/85580 (PMC13037756; doi:10.2196/85580)
Supplement: Multimedia Appendix 1 [file medinform-v14-e85580-s001.docx]

# **Supplementary A**

Note Buddy's architecture utilizes Microsoft Azure Speech Services for speech recognition and OpenAI's GPT-4o model hosted on Singapore's Healthcare Commercial Cloud infrastructure. The speech recognition pipeline is able to detect the language used at the word level, enabling accurate transcription of conversations where code-switching between languages occur, something common in Singapore. Audio streams are processed in real-time with speaker diarization to distinguish between clinician and patient voices. Privacy protection is enforced through automated detection and masking of personally identifiable information (names, identification numbers, contact details) before any data reaches the language model.


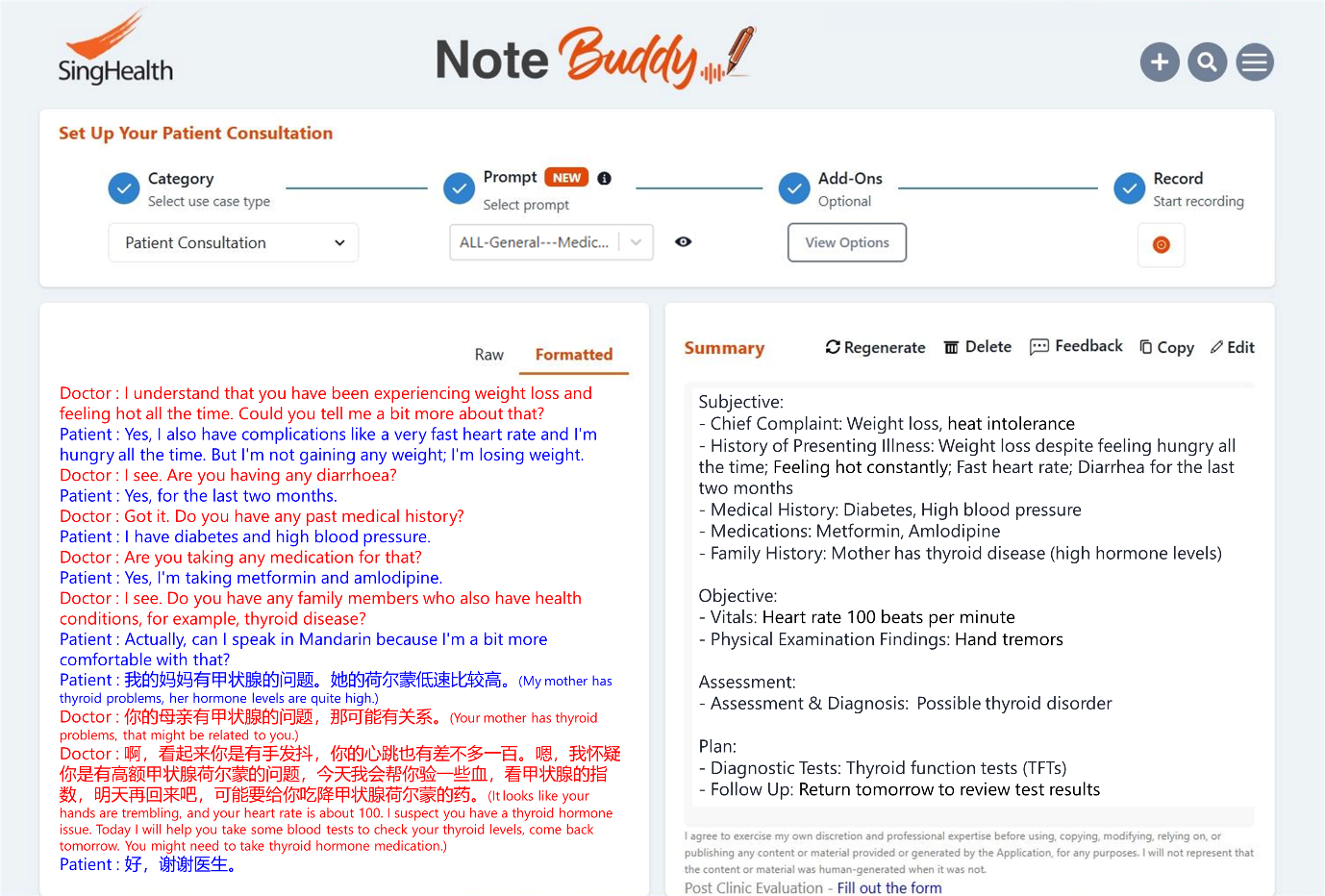


**Figure S1:** Screenshot of the Note Buddy user interface. The window on the left is the Formatted Transcript between the clinician and patient. The right window is the output panel where the summary of the consultation is generated based on the prompt selected.

**Figure S2:** Screenshot of the Note Buddy Prompt Library. Users will be able to view created and shared prompts by other users of Note Buddy. Users can also favourite prompts, by clicking on the star, making it easy for them to use these prompts for future Note Buddy use.
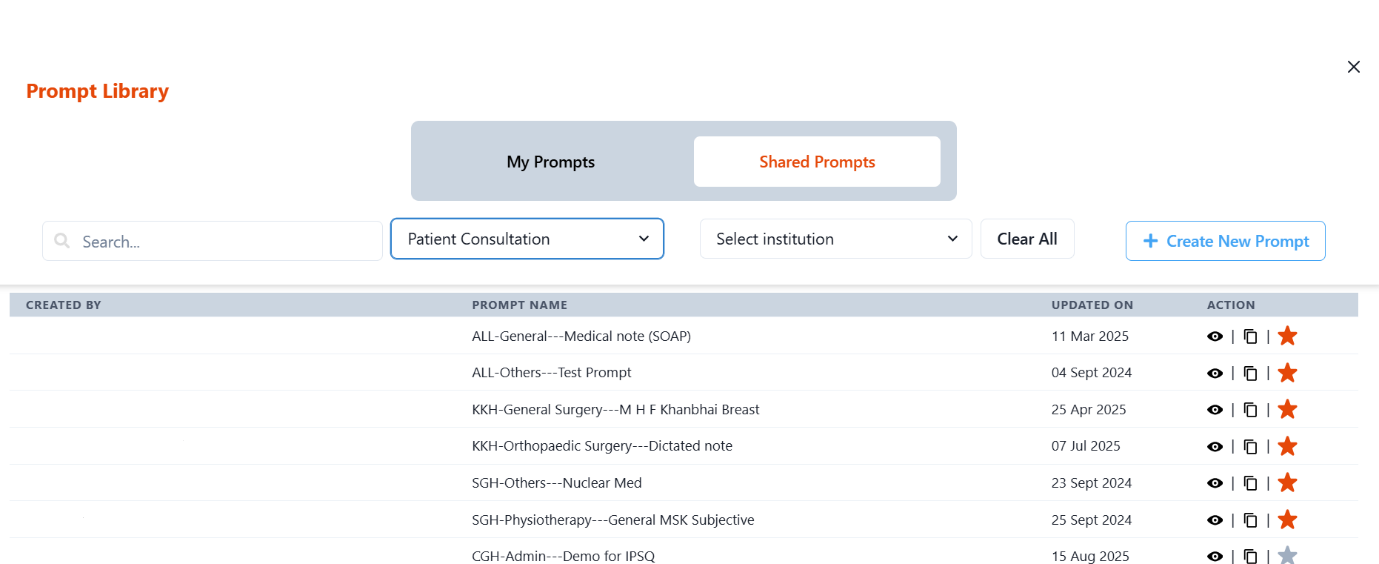


**Supplementary B**
The study team designed a macro-enabled Excel sheet to enable observers to collect time-motion study data in a consistent and standardised manner. The observer clicks “*Patient Enters*” when the patient enters the consultation room and clicks “*Toggle Eye Contact*” whenever the clinician shifts between documenting and making eye contact. At the end of the consultation, “*Patient Leaves Clinic*” is clicked as the patient exits. If the clinician continues documenting after the patient leaves, the observer clicks “End Documentation” once the documentation is completed.


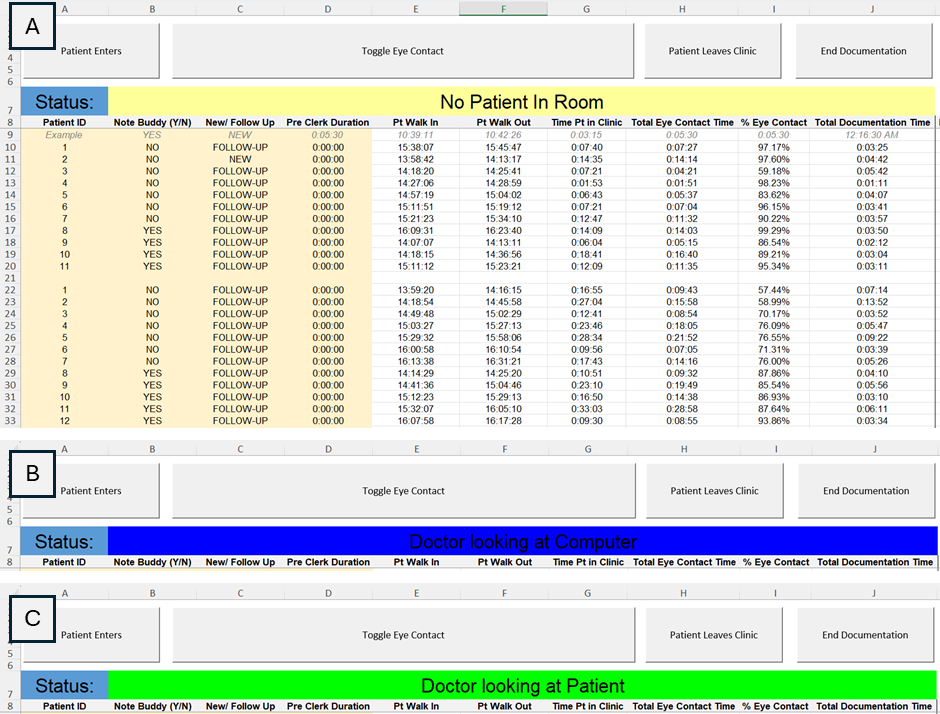


**Figure S3**: Screenshots of the Macro-Enabled Excel sheet used for the Time Motion Study. Figure S3-B and S3-C shows the interface and status when the Doctor is documenting and making eye-contact with the patient respectively.

**Supplementary C**

Patient survey questions

1. What is your age?
   1. 0-19
   2. 20-29
   3. 30-39
   4. 40-49
   5. 50-59
   6. 60-64
   7. 65 and above
2. Preferred Language
   1. English
   2. Chinese & dialect
   3. Malay
   4. Tamil
   5. Others
3. Did you feel your doctor spent enough time engaging you / listening and talking to you when the AI scribe was helping to take notes?
   1. Yes, the doctor focused on me more
   2. About the same as before
   3. No, can be better
   4. Unsure / I don’t know
   5. This is my first visit with the doctor
4. Overall, do you feel that your consultation was enhanced by the use of an AI scribe?
   1. Much Better
   2. Somewhat Better
   3. No Effect
   4. Worse
5. Do you feel comfortable with an AI tool being used in the consultation?
   1. Very Comfortable
   2. Comfortable
   3. Neutral
   4. Uncomfortable
   5. Very uncomfortable
6. Would you recommend that other doctors use this AI scribe in their consultations?
   1. Yes
   2. Neutral
   3. No
7. Is there anything else you would like to tell us about your experience with the AI scribe during this visit?

Survey Response Coding

For analysis, survey responses were coded into three categories (Yes/Neutral/No) as follows:

**Question 3 (Doctor engagement):**

- "Yes, the doctor focused on me more" = Yes
- "About the same as before", "Unsure / I don't know", "This is my first visit with the doctor" = Neutral
- "No, can be better" = No

**Question 4 (Consultation enhancement):**

- "Much Better" and "Somewhat Better" = Yes
- "No Effect" = Neutral
- "Worse" = No

**Question 5 (Comfort with AI tool):**

- "Very Comfortable" and "Comfortable" = Yes
- "Neutral" = Neutral
- "Uncomfortable" and "Very uncomfortable" = No.

**Question 6 (Recommendation):**

- "Yes" = Yes
- "Neutral" = Neutral
- "No" = No.
